# 1-(5-Bromo-2-oxoindolin-3-yl­idene)-4-phenyl­thio­semicarbazide

**DOI:** 10.1107/S1600536813020497

**Published:** 2013-07-27

**Authors:** Katlen C. T. Bandeira, Leandro Bresolin, Christian Näther, Inke Jess, Adriano Bof de Oliveira

**Affiliations:** a Escola de Química e Alimentos, Universidade Federal do Rio Grande, Av. Itália km 08, Campus Carreiros, 96203-903 Rio Grande, RS, Brazil; b Institut für Anorganische Chemie, Christian-Albrechts-Universität zu Kiel, Max-Eyth Strasse 2, D-24118 Kiel, Germany; c Departamento de Química, Universidade Federal de Sergipe, Av. Marechal Rondon s/n, Campus, 49100-000 São Cristóvão, SE, Brazil

## Abstract

In the title compound, C_15_H_11_BrN_4_OS, the least-squares plane through the 5-bromo­isatin fragment forms a dihedral angle of 13.63 (14)° with the phenyl ring. The mol­ecular conformation features intra­molecular N—H⋯N and N—H⋯O hydrogen bonds. In the crystal, mol­ecules are connected *via* pairs of N—H⋯O inter­actions into centrosymmetric dimers. Additionally, π–π stacking inter­actions link mol­ecules into chains parallel to the *a* axis with short C⋯C distances being observed between the phenyl and thio­carbonyl [3.236 (8) Å] groups and between the thio­carbonyl and carbonyl [3.351 (4) Å] groups of stacked mol­ecules.

## Related literature

For the pharmacological properties of isatin-thio­semi­carbazone derivatives against cruzain, falcipain-2 and rhodesain, see: Chiyanzu *et al.* (2003 ▶). For the synthesis of 5-bromo­isatin-3-thio­semicarbazone, see: Campaigne & Archer (1952 ▶). For the crystal structure of 1-(5-bromo-2-oxoindolin-3-yl­idene)thio­semicarbazide aceto­nitrile monosolvate, see: Pederzolli *et al.* (2011 ▶).
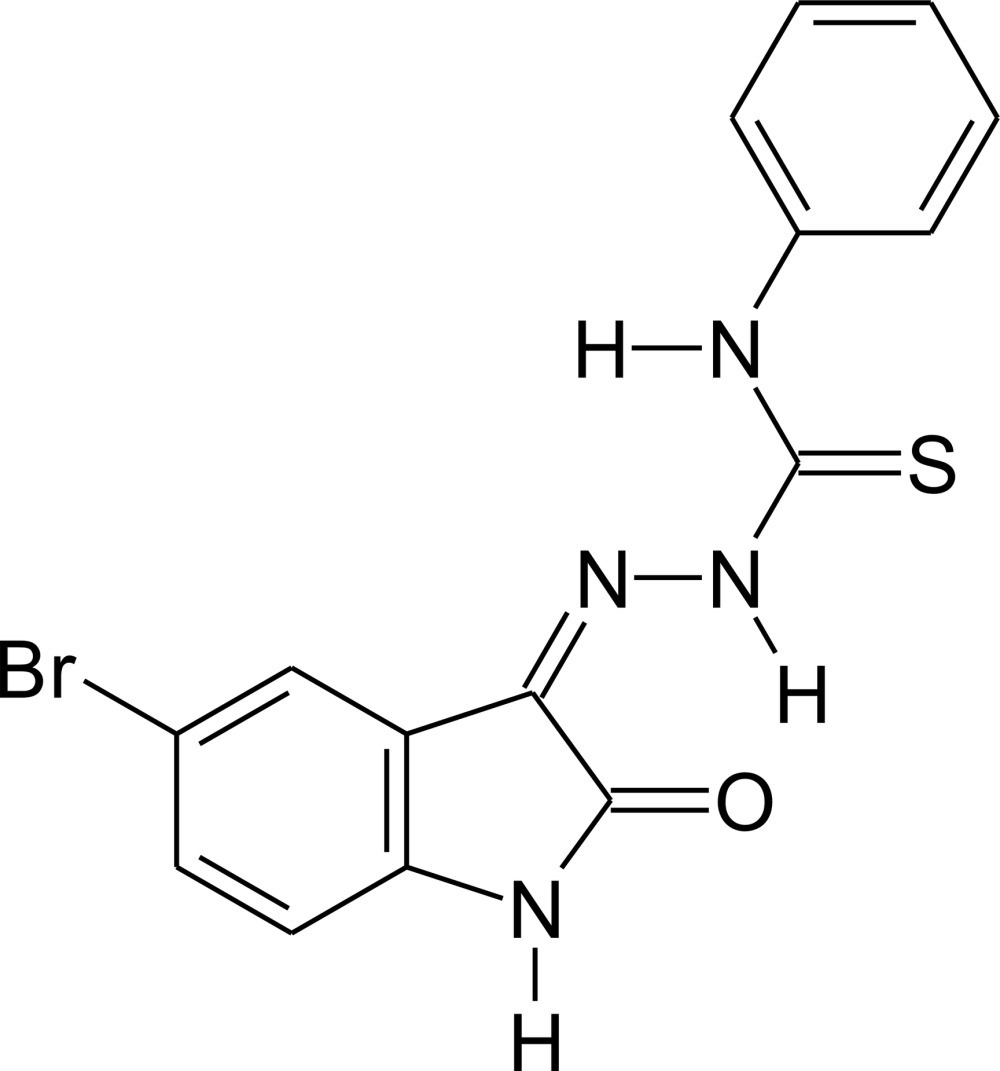


## Experimental

### 

#### Crystal data

C_15_H_11_BrN_4_OS
*M* *_r_* = 375.25
Monoclinic, 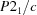

*a* = 5.6882 (3) Å
*b* = 18.4086 (9) Å
*c* = 14.4668 (10) Å
β = 91.272 (8)°
*V* = 1514.47 (15) Å^3^
*Z* = 4
Mo *K*α radiation
μ = 2.86 mm^−1^
*T* = 200 K
0.12 × 0.10 × 0.08 mm

#### Data collection

Stoe IPDS-1 diffractometer
Absorption correction: numerical (*X-SHAPE* and *X-RED32*; Stoe & Cie, 2008 ▶) *T*_min_ = 0.633, *T*_max_ = 0.677
13502 measured reflections
2903 independent reflections
2235 reflections with *I* > 2σ(*I*)
*R*_int_ = 0.064

#### Refinement

*R*[*F* ^2^ > 2σ(*F* ^2^)] = 0.046
*wR*(*F* ^2^) = 0.105
*S* = 1.04
2903 reflections
199 parameters
H-atom parameters constrained
Δρ_max_ = 0.67 e Å^−3^
Δρ_min_ = −1.11 e Å^−3^

### 

Data collection: *X-AREA* (Stoe & Cie, 2008 ▶); cell refinement: *X-AREA*; data reduction: *X-RED32* (Stoe & Cie, 2008 ▶); program(s) used to solve structure: *SHELXS97* (Sheldrick, 2008 ▶); program(s) used to refine structure: *SHELXL97* (Sheldrick, 2008 ▶); molecular graphics: *DIAMOND* (Brandenburg, 2006 ▶); software used to prepare material for publication: *publCIF* (Westrip, 2010 ▶).

## Supplementary Material

Crystal structure: contains datablock(s) I, publication_text. DOI: 10.1107/S1600536813020497/fy2102sup1.cif

Structure factors: contains datablock(s) I. DOI: 10.1107/S1600536813020497/fy2102Isup2.hkl

Supplementary material file. DOI: 10.1107/S1600536813020497/fy2102Isup3.cml

Additional supplementary materials:  crystallographic information; 3D view; checkCIF report

## Figures and Tables

**Table 1:** Hydrogen-bond geometry (Å, °)

*D*—H⋯*A*	*D*—H	H⋯*A*	*D*⋯*A*	*D*—H⋯*A*
N1—H1⋯O1^i^	0.88	2.00	2.858 (3)	166
N3—H3⋯O1	0.88	2.07	2.762 (3)	135
N4—H4*A*⋯N2	0.88	2.16	2.613 (4)	112

Symmetry code: (i) 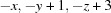.

**Fig. 1.**
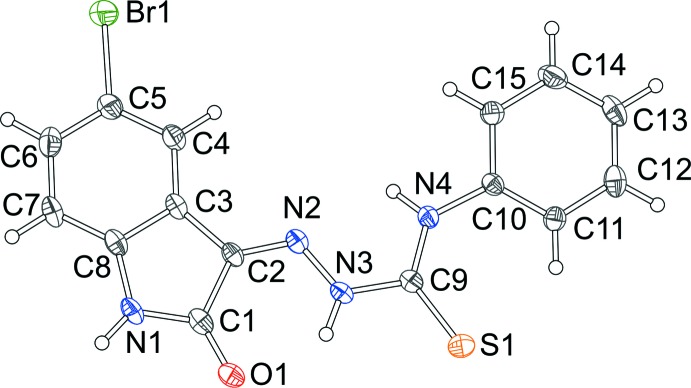
The molecular structure of the title compound with labeling and displacement ellipsoids drawn at the 40% probability level.

**Fig. 2.**
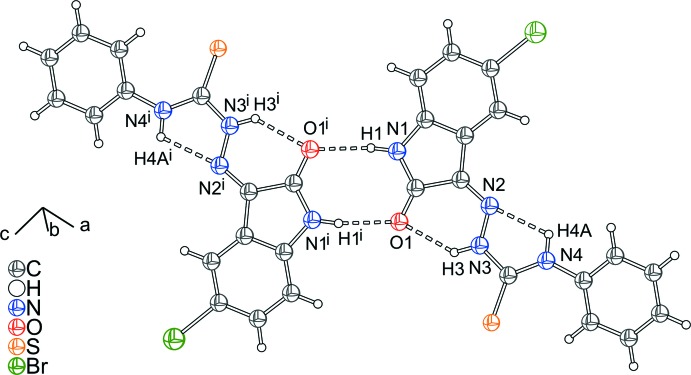
Molecules of the title compound connected through inversion centers via pairs of N—H···O interactions. Intramolecular N—H···N and N—H···O hydrogen bonds are also shown. H-interactions are indicated as dashed lines and the Figure is simplified for clarity. Symmetry code: (i)-x,-y + 1,-z + 3.

**Fig. 3.**
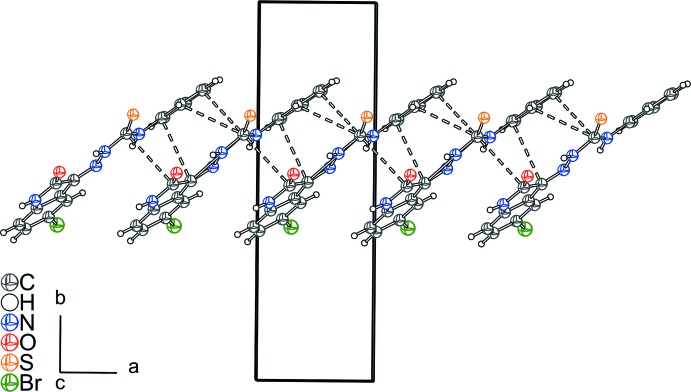
Crystal structure of the title compound in a view along the crystallographic c-axis. The π–π-interactions are drawn as dashed lines, highlighting C···C distances ranging from 3.236 (8) to 3.471 (7) Å. The molecular arrangment in layers, stacked into the direction of the crystallographic a-axis, is simplified for clarity.

## supplementary crystallographic information

### Comment

Thiosemicarbazone derivatives have a wide range of biological properties. For example, isatin-based synthetic thiosemicarbazones show pharmacological activity against cruzain, falcipain-2 and rhodesain (Chiyanzu *et al.*, 2003). As part of our study of thiosemicarbazone derivatives, we report herein the crystal structure of 5-bromoisatin-3-(4-phenyl)thiosemicarbazone. In the title compound, in which the molecular structure matches the asymmetric unit, the maximal deviation from the least squares plane through all non-hydrogen atoms amounts to 0.2917 (33) Å for C14. The molecule shows an *E* conformation for the atoms about the N2—N3 bond (Fig. 1). The *E* conformation for the thiosemicarbazone fragment is also observed in the crystal structure of the 5-bromoisatin-3-thiosemicarbazone acetonitrile monosolvate (Pederzolli *et al.*, 2011) and is related with the intramolecular N—H···N and N—H···O hydrogen-bonding interactions (Fig. 1; Table 1). The mean deviations from the least squares planes for the 5-bromoisatin, C1—C8/Br1/O1 and the terminal aromatic ring, C10—C15, fragments amounts to 0.0459 (19) Å for O1 and 0.0032 (22) Å for C10, respectively, and the dihedral angle between the two planes is 13.63 (14)°. The molecules are connected *via* centrosymmetric pairs of N—H···O interactions (Fig. 2; Table 1). Additionally, π–π-interactions are observed, with C···C distances of 3.236 (8), 3.351 (4), 3.451 (5) and 3.471 (7) Å. The molecules are arranged in layers and are stacked into the direction of the crystallographic *a*-axis (Fig. 3).

### Experimental

The starting materials were commercially available and were used without further purification. The 5-bromoisatine-3-(4-phenyl)thiosemicarbazone synthesis was adapted from a procedure reported previously (Campaigne & Archer, 1952). The hydrochloric acid catalyzed reaction of 5-bromoisatin (8.83 mmol) and (4-phenyl)thiosemicarbazide (8.83 mmol) in a 1:1 mixture of ethanol and water (50 ml) was refluxed for 6 h. After cooling and filtering, the title compound was obtained. Crystals suitable for X-ray diffraction of the title compound were obtained by the slow evaporation of the solvents.

### Refinement

All C—H and N—H H atoms were located in difference map, but were positioned with idealized geometry and were refined isotropically with *U*_iso_(H) = 1.2 *U*_eq_(C, N) using a riding model with C—H = 0.93 Å for aromatic and N—H = 0.88 Å for N-bound H atoms.

### Crystal data

**Table:**

C_15_H_11_BrN_4_OS	*F*(000) = 752
*M**_r_* = 375.25	*D*_x_ = 1.646 Mg m^−^^3^
Monoclinic, *P*2_1_/*c*	Mo *K*α radiation, λ = 0.71073 Å
Hall symbol: -P 2ybc	Cell parameters from 13698 reflections
*a* = 5.6882 (3) Å	θ = 2.6–26.0°
*b* = 18.4086 (9) Å	µ = 2.86 mm^−^^1^
*c* = 14.4668 (10) Å	*T* = 200 K
β = 91.272 (8)°	Block, yellow
*V* = 1514.47 (15) Å^3^	0.12 × 0.10 × 0.08 mm
*Z* = 4	

### Data collection

**Table:**

Stoe IPDS-1 diffractometer	2903 independent reflections
Radiation source: fine-focus sealed tube, Stoe IPDS-1	2235 reflections with *I* > 2σ(*I*)
Graphite monochromator	*R*_int_ = 0.064
φ scans	θ_max_ = 26.0°, θ_min_ = 2.6°
Absorption correction: numerical (*X-SHAPE* and *X-RED32*; Stoe & Cie, 2008)	*h* = −6→6
*T*_min_ = 0.633, *T*_max_ = 0.677	*k* = −22→22
13502 measured reflections	*l* = −17→17

### Refinement

**Table:**

Refinement on *F*^2^	Primary atom site location: structure-invariant direct methods
Least-squares matrix: full	Secondary atom site location: difference Fourier map
*R*[*F*^2^ > 2σ(*F*^2^)] = 0.046	Hydrogen site location: inferred from neighbouring sites
*wR*(*F*^2^) = 0.105	H-atom parameters constrained
*S* = 1.04	*w* = 1/[σ^2^(*F*_o_^2^) + (0.0517*P*)^2^ + 1.4796*P*] where *P* = (*F*_o_^2^ + 2*F*_c_^2^)/3
2903 reflections	(Δ/σ)_max_ = 0.001
199 parameters	Δρ_max_ = 0.67 e Å^−^^3^
0 restraints	Δρ_min_ = −1.11 e Å^−^^3^

### Special details

**Table:**

Geometry. All e.s.d.'s (except the e.s.d. in the dihedral angle between two l.s. planes) are estimated using the full covariance matrix. The cell e.s.d.'s are taken into account individually in the estimation of e.s.d.'s in distances, angles and torsion angles; correlations between e.s.d.'s in cell parameters are only used when they are defined by crystal symmetry. An approximate (isotropic) treatment of cell e.s.d.'s is used for estimating e.s.d.'s involving l.s. planes.
Refinement. Refinement of *F*^2^ against ALL reflections. The weighted *R*-factor *wR* and goodness of fit *S* are based on *F*^2^, conventional *R*-factors *R* are based on *F*, with *F* set to zero for negative *F*^2^. The threshold expression of *F*^2^ > σ(*F*^2^) is used only for calculating *R*-factors(gt) *etc*. and is not relevant to the choice of reflections for refinement. *R*-factors based on *F*^2^ are statistically about twice as large as those based on *F*, and *R*- factors based on ALL data will be even larger.

### Fractional atomic coordinates and isotropic or equivalent isotropic displacement parameters (Å^2^)

**Table:**

	*x*	*y*	*z*	*U*_iso_*/*U*_eq_	
N1	0.0836 (5)	0.47322 (14)	1.38606 (19)	0.0312 (6)	
H1	−0.0271	0.4583	1.4231	0.037*	
C1	0.2595 (5)	0.51911 (16)	1.4116 (2)	0.0283 (7)	
O1	0.2888 (4)	0.54902 (12)	1.48762 (15)	0.0316 (5)	
C2	0.4124 (5)	0.52739 (15)	1.3285 (2)	0.0247 (6)	
C3	0.3024 (5)	0.48403 (15)	1.2553 (2)	0.0261 (6)	
C4	0.3560 (6)	0.47236 (17)	1.1638 (2)	0.0324 (7)	
H4	0.4915	0.4934	1.1374	0.039*	
C5	0.2042 (7)	0.42873 (18)	1.1120 (2)	0.0374 (8)	
Br1	0.28023 (10)	0.40737 (3)	0.98769 (3)	0.0688 (2)	
C6	0.0060 (6)	0.39716 (18)	1.1491 (3)	0.0381 (8)	
H6	−0.0934	0.3675	1.1113	0.046*	
C7	−0.0482 (6)	0.40850 (17)	1.2412 (3)	0.0351 (8)	
H7	−0.1826	0.3868	1.2675	0.042*	
C8	0.1004 (5)	0.45251 (16)	1.2930 (2)	0.0283 (7)	
N2	0.6012 (4)	0.56591 (13)	1.32193 (18)	0.0256 (5)	
N3	0.6730 (5)	0.60375 (13)	1.39722 (17)	0.0260 (5)	
H3	0.6004	0.5976	1.4497	0.031*	
C9	0.8582 (5)	0.65192 (15)	1.3933 (2)	0.0245 (6)	
S1	0.91317 (16)	0.70212 (5)	1.48665 (6)	0.0369 (2)	
N4	0.9676 (4)	0.65094 (13)	1.31167 (17)	0.0260 (5)	
H4A	0.9123	0.6186	1.2721	0.031*	
C10	1.1570 (5)	0.69326 (15)	1.2785 (2)	0.0241 (6)	
C11	1.3184 (5)	0.72936 (16)	1.3353 (2)	0.0270 (6)	
H11	1.3035	0.7283	1.4005	0.032*	
C12	1.5030 (6)	0.76727 (17)	1.2954 (3)	0.0348 (7)	
H12	1.6135	0.7922	1.3340	0.042*	
C13	1.5277 (6)	0.76906 (19)	1.2009 (3)	0.0373 (8)	
H13	1.6537	0.7952	1.1745	0.045*	
C14	1.3681 (6)	0.7327 (2)	1.1451 (2)	0.0410 (8)	
H14	1.3847	0.7335	1.0799	0.049*	
C15	1.1830 (6)	0.6949 (2)	1.1832 (2)	0.0355 (8)	
H15	1.0736	0.6700	1.1440	0.043*	

### Atomic displacement parameters (Å^2^)

**Table:**

	*U*^11^	*U*^22^	*U*^33^	*U*^12^	*U*^13^	*U*^23^
N1	0.0231 (15)	0.0324 (14)	0.0385 (15)	−0.0034 (10)	0.0124 (11)	0.0030 (11)
C1	0.0216 (17)	0.0269 (15)	0.0367 (18)	0.0058 (11)	0.0065 (13)	0.0074 (12)
O1	0.0272 (13)	0.0369 (12)	0.0312 (12)	0.0018 (9)	0.0096 (9)	0.0014 (9)
C2	0.0189 (16)	0.0248 (14)	0.0307 (16)	0.0013 (11)	0.0065 (12)	0.0032 (11)
C3	0.0218 (17)	0.0222 (14)	0.0344 (17)	−0.0003 (11)	0.0059 (12)	0.0043 (12)
C4	0.0320 (19)	0.0305 (16)	0.0350 (18)	−0.0055 (13)	0.0058 (14)	0.0036 (13)
C5	0.047 (2)	0.0325 (17)	0.0329 (18)	−0.0087 (15)	0.0038 (15)	0.0041 (13)
Br1	0.1019 (4)	0.0700 (3)	0.0350 (2)	−0.0476 (3)	0.0120 (2)	−0.00818 (19)
C6	0.036 (2)	0.0311 (17)	0.046 (2)	−0.0080 (14)	−0.0056 (15)	0.0015 (14)
C7	0.0242 (18)	0.0318 (16)	0.050 (2)	−0.0061 (13)	0.0075 (14)	0.0040 (14)
C8	0.0219 (17)	0.0252 (14)	0.0380 (18)	0.0012 (11)	0.0067 (13)	0.0059 (12)
N2	0.0233 (14)	0.0235 (12)	0.0300 (14)	0.0007 (10)	0.0042 (10)	0.0025 (10)
N3	0.0251 (14)	0.0289 (13)	0.0245 (13)	−0.0019 (10)	0.0072 (10)	0.0029 (10)
C9	0.0223 (16)	0.0254 (14)	0.0259 (15)	0.0028 (11)	0.0025 (12)	0.0038 (11)
S1	0.0411 (5)	0.0419 (5)	0.0279 (4)	−0.0053 (4)	0.0071 (3)	−0.0087 (3)
N4	0.0242 (14)	0.0281 (12)	0.0260 (13)	−0.0050 (10)	0.0048 (10)	−0.0026 (10)
C10	0.0208 (16)	0.0257 (14)	0.0258 (15)	0.0011 (11)	0.0024 (11)	0.0032 (11)
C11	0.0240 (17)	0.0273 (15)	0.0297 (16)	0.0001 (12)	−0.0003 (12)	−0.0007 (12)
C12	0.0236 (18)	0.0298 (16)	0.051 (2)	−0.0014 (12)	−0.0024 (14)	0.0018 (14)
C13	0.0224 (18)	0.0391 (18)	0.051 (2)	−0.0013 (13)	0.0071 (15)	0.0145 (15)
C14	0.031 (2)	0.062 (2)	0.0303 (18)	−0.0024 (16)	0.0070 (14)	0.0084 (16)
C15	0.0253 (18)	0.053 (2)	0.0278 (17)	−0.0086 (15)	0.0007 (13)	−0.0007 (14)

### Geometric parameters (Å, º)

**Table:**

N1—C1	1.355 (4)	N3—C9	1.379 (4)
N1—C8	1.405 (4)	N3—H3	0.8800
N1—H1	0.8800	C9—N4	1.347 (4)
C1—O1	1.238 (4)	C9—S1	1.660 (3)
C1—C2	1.507 (4)	N4—C10	1.421 (4)
C2—N2	1.292 (4)	N4—H4A	0.8800
C2—C3	1.456 (4)	C10—C11	1.388 (4)
C3—C4	1.382 (5)	C10—C15	1.391 (4)
C3—C8	1.408 (4)	C11—C12	1.397 (5)
C4—C5	1.387 (5)	C11—H11	0.9500
C4—H4	0.9500	C12—C13	1.378 (5)
C5—C6	1.387 (5)	C12—H12	0.9500
C5—Br1	1.899 (4)	C13—C14	1.375 (5)
C6—C7	1.390 (5)	C13—H13	0.9500
C6—H6	0.9500	C14—C15	1.386 (5)
C7—C8	1.380 (5)	C14—H14	0.9500
C7—H7	0.9500	C15—H15	0.9500
N2—N3	1.349 (4)		
			
C1—N1—C8	111.4 (3)	N2—N3—C9	121.1 (2)
C1—N1—H1	124.3	N2—N3—H3	119.4
C8—N1—H1	124.3	C9—N3—H3	119.4
O1—C1—N1	127.1 (3)	N4—C9—N3	113.3 (3)
O1—C1—C2	126.5 (3)	N4—C9—S1	129.7 (2)
N1—C1—C2	106.3 (3)	N3—C9—S1	117.0 (2)
N2—C2—C3	126.3 (3)	C9—N4—C10	131.0 (3)
N2—C2—C1	127.5 (3)	C9—N4—H4A	114.5
C3—C2—C1	106.2 (3)	C10—N4—H4A	114.5
C4—C3—C8	120.4 (3)	C11—C10—C15	119.5 (3)
C4—C3—C2	133.0 (3)	C11—C10—N4	124.0 (3)
C8—C3—C2	106.6 (3)	C15—C10—N4	116.4 (3)
C3—C4—C5	117.4 (3)	C10—C11—C12	119.2 (3)
C3—C4—H4	121.3	C10—C11—H11	120.4
C5—C4—H4	121.3	C12—C11—H11	120.4
C6—C5—C4	122.3 (3)	C13—C12—C11	121.1 (3)
C6—C5—Br1	118.9 (3)	C13—C12—H12	119.5
C4—C5—Br1	118.6 (3)	C11—C12—H12	119.5
C5—C6—C7	120.5 (3)	C14—C13—C12	119.3 (3)
C5—C6—H6	119.7	C14—C13—H13	120.3
C7—C6—H6	119.7	C12—C13—H13	120.3
C8—C7—C6	117.5 (3)	C13—C14—C15	120.6 (3)
C8—C7—H7	121.2	C13—C14—H14	119.7
C6—C7—H7	121.2	C15—C14—H14	119.7
C7—C8—N1	128.8 (3)	C14—C15—C10	120.2 (3)
C7—C8—C3	121.8 (3)	C14—C15—H15	119.9
N1—C8—C3	109.4 (3)	C10—C15—H15	119.9
C2—N2—N3	117.5 (3)		
			
C8—N1—C1—O1	177.1 (3)	C4—C3—C8—C7	1.0 (5)
C8—N1—C1—C2	−2.2 (3)	C2—C3—C8—C7	179.0 (3)
O1—C1—C2—N2	1.1 (5)	C4—C3—C8—N1	−179.2 (3)
N1—C1—C2—N2	−179.6 (3)	C2—C3—C8—N1	−1.2 (3)
O1—C1—C2—C3	−177.9 (3)	C3—C2—N2—N3	178.6 (3)
N1—C1—C2—C3	1.5 (3)	C1—C2—N2—N3	−0.2 (4)
N2—C2—C3—C4	−1.5 (5)	C2—N2—N3—C9	−173.3 (3)
C1—C2—C3—C4	177.5 (3)	N2—N3—C9—N4	−6.0 (4)
N2—C2—C3—C8	−179.2 (3)	N2—N3—C9—S1	173.5 (2)
C1—C2—C3—C8	−0.2 (3)	N3—C9—N4—C10	177.3 (3)
C8—C3—C4—C5	−0.2 (5)	S1—C9—N4—C10	−2.2 (5)
C2—C3—C4—C5	−177.6 (3)	C9—N4—C10—C11	21.9 (5)
C3—C4—C5—C6	−0.3 (5)	C9—N4—C10—C15	−161.0 (3)
C3—C4—C5—Br1	−176.8 (2)	C15—C10—C11—C12	0.6 (4)
C4—C5—C6—C7	0.1 (6)	N4—C10—C11—C12	177.7 (3)
Br1—C5—C6—C7	176.6 (3)	C10—C11—C12—C13	−0.3 (5)
C5—C6—C7—C8	0.6 (5)	C11—C12—C13—C14	−0.2 (5)
C6—C7—C8—N1	179.0 (3)	C12—C13—C14—C15	0.4 (5)
C6—C7—C8—C3	−1.2 (5)	C13—C14—C15—C10	−0.1 (6)
C1—N1—C8—C7	−178.0 (3)	C11—C10—C15—C14	−0.4 (5)
C1—N1—C8—C3	2.2 (3)	N4—C10—C15—C14	−177.7 (3)

### Hydrogen-bond geometry (Å, º)

**Table:**

*D*—H···*A*	*D*—H	H···*A*	*D*···*A*	*D*—H···*A*
N1—H1···O1^i^	0.88	2.00	2.858 (3)	166
N3—H3···O1	0.88	2.07	2.762 (3)	135
N4—H4*A*···N2	0.88	2.16	2.613 (4)	112

Symmetry code: (i) −*x*, −*y*+1, −*z*+3.
